# A Biomedically Enriched Collection of 7000 Human ORF Clones

**DOI:** 10.1371/journal.pone.0001528

**Published:** 2008-01-30

**Authors:** Andreas Rolfs, Yanhui Hu, Lars Ebert, Dietmar Hoffmann, Dongmei Zuo, Niro Ramachandran, Jacob Raphael, Fontina Kelley, Seamus McCarron, Daniel A. Jepson, Binghua Shen, Munira M. A. Baqui, Joseph Pearlberg, Elena Taycher, Craig DeLoughery, Andreas Hoerlein, Bernhard Korn, Joshua LaBaer

**Affiliations:** 1 Harvard Institute of Proteomics, Harvard Medical School, Cambridge, Massachusetts, United States of America; 2 Deutsches Ressourcenzentrum fuer Genomforschung (RZPD), Heidelberg, Germany; 3 Sanofi-Aventis, Cambridge, Massachusetts, United States of America; Fred Hutchinson Cancer Research Center, United States of America

## Abstract

We report the production and availability of over 7000 fully sequence verified plasmid ORF clones representing over 3400 unique human genes. These ORF clones were derived using the human MGC collection as template and were produced in two formats: with and without stop codons. Thus, this collection supports the production of either native protein or proteins with fusion tags added to either or both ends. The template clones used to generate this collection were enriched in three ways. First, gene redundancy was removed. Second, clones were selected to represent the best available GenBank reference sequence. Finally, a literature-based software tool was used to evaluate the list of target genes to ensure that it broadly reflected biomedical research interests. The target gene list was compared with 4000 human diseases and over 8500 biological and chemical MeSH classes in ∼15 Million publications recorded in PubMed at the time of analysis. The outcome of this analysis revealed that relative to the genome and the MGC collection, this collection is enriched for the presence of genes with published associations with a wide range of diseases and biomedical terms without displaying a particular bias towards any single disease or concept. Thus, this collection is likely to be a powerful resource for researchers who wish to study protein function in a set of genes with documented biomedical significance.

## Introduction

The study of protein function often demands high quality plasmid clones that contain the relevant open reading frames (ORFs) in a format compatible with protein expression. Increasingly, high throughput methods have created the demand for clones that encode a class of proteins of interest or the entire proteome of a species. Functional studies rely on *in vivo* expression for phenotypic studies or expression and purification by various means for biochemical analysis. Utilizing recombinational cloning vectors and including only the coding sequences, with all untranslated sequences removed, ensures maximum flexibility, including protein expression in a broad experimental range with various tagging options for either end of the protein. In addition, to avoid erroneous or ambiguous results regarding the expressed proteins, it is important that the plasmids are clonal isolates that are fully sequence verified.

For many eukaryotic species, including humans, the number of protein coding sequences exceeds 15,000 genes, making the production of comprehensive sequence-verified ORF clone collections daunting and expensive. In fact, a complete set of source material for expressed genes in humans does not yet exist [1]–[3]. One strategy is for researchers to focus on (a) meaningful subset(s) of genes for functional studies relevant to the biological questions they wish to address. For a human ORF collection the criteria for selecting genes are mostly driven by researchers' interest and clone availability, resulting often in either collections of special interest [4] [5], or more ‘random’ lists of genes in collections (RZPD, Invitrogen).

In recent years, a publicly funded project, the Mammalian Gene Collection (MGC), aimed to create for multiple species, but especially for man and mouse, collections of well annotated, fully sequence validated cDNA clones [6]. However, the MGC clones cannot easily be employed directly in functional proteomics experiments because they are in many different vector backbones and contain 5′ and 3′ untranslated sequences. On the other hand, because they are fully sequenced and well annotated, these clones provide an excellent starting point for creating ORF clones. At least one such ORF set has been made so far, although that set comprises pools of clones that are not sequence verified [7] [8] and thus has potential ambiguity. Currently, there are also four human ORF collections available from commercial distributors that were clonally isolated and at least partially sequence validated. The recently created ORFeome Collaboration (http://www.orfeomecollaboration.org/) [9] is a project planned to bring to all researchers an ORF clone collection that provides at least one representative ORF clone for all human genes, similar in quality and scope to the MGC clones, with all clones being fully sequence validated.

A limitation of the recombinational cloning vectors used for these ORF clones is that each clone must be committed to one of two non-interchangeable formats: *closed* (with stop codon; can express native protein) or *fusion* (no stop codon; enables the addition of carboxyl-terminal fusion peptides). As each format has unique advantages not available for the other, the ideal collection would include both.

Previously we reported the production of two smaller human clone sets in the Creator™ system. One set focused on kinase genes, both well-studied as well as novel or hypothetical ones [5]; the other clone set covered over 1000 genes associated with breast cancer [10], identified in publications using software developed in our group [11]. Here we report the production and complete sequence validation of 7000 clones, HLFEX7000 (>3400 unique human genes in two formats), and the distribution of these genes with respect to their relationship to disease and biological terms in publications in PubMed.

## Results

### Gene Selection

To make the most useful ORF clone set of the MGC clones, we wished to select an enriched set of genes that is of particular interest to both medicine and biology. In addition, we wished to exclude clones that corresponded to partial gene products and to eliminate redundancy. We first excluded the subset of all MGC clones where the CDS length was less than 90% of the length of the longest corresponding NCBI RefSeq sequence [12]–[14]. We then removed redundancy within the MGC clone set, and picked the clone closest to the longest reference sequence by CDS length as a best MGC representative for each gene. This reduced the number of candidate template clones from 13493 to 7992, representing 7992 genes.

**Figure 1:**
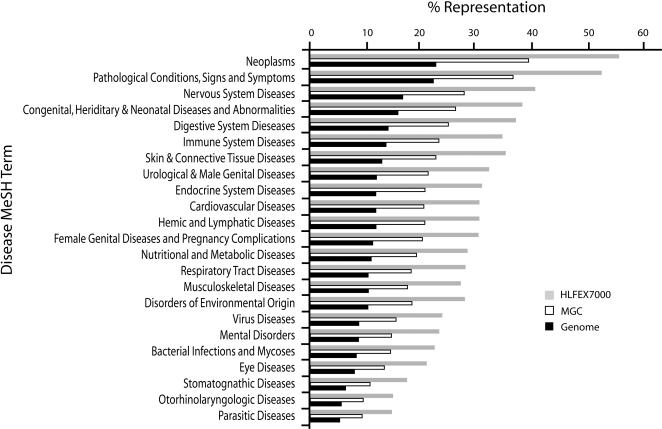
Genes associated with Disease Classes (MeSH) in Publications. The clone target list (HFLEX7000) was compared with all human genes (EntrezGene, 2004) and all genes represented by MGC (2004) with respect to published relationships of the genes to human diseases. The targeted genes reveal similar proportionality to the other gene lists but a general enrichment of genes related to diseases (Table 1; Supplementary Table S2).

Our discussions with researchers indicated that a focused set of genes in both formats (closed and fusion) would be of more value that a large set in only one format. To ensure that our final gene set (Supplementary Table S1) was enriched for genes related to human diseases without any specific bias, the candidate list was used to query MedGene [11] for genes associated with about 4000 human diseases. As described, MedGene is an automated literature-mining tool, which comprehensively summarizes and estimates the relative strengths of all human gene-disease relationships reported in Medline/PubMed. The result of this query was compared with queries using either all unique genes represented in MGC or all ∼33,000 human genes listed at the time in LocusLink (2004, now: EntrezGene [15]). As shown for a subset of diseases in Figure 1; Table 1 (complete list: Supplementary Table S2), the resulting target list: (a) was highly enriched for the presence of genes with published associations with a wide range of human diseases; (b) had a similar relative ratio among the various diseases to that of both the genome and the MGC; and (c) displayed a broad overlap among different diseases allowing multiple diseases to be addressed with this set of ORF clones.

**Table 1:** MeSH Term Analysis for Gene and Diseases Association in Publications; Examples

Disease MeSH Term	Genome		MGC		HFLEX7000		MGC/Genome	HFLEX7000/Genome	HFLEX7000/MGC
	#	(%)	#	(%)	#	(%)			
Neoplasms	7614	23.0	3904	40.0	1884	56.4	1.74	2.45	1.41
Pathological Conditions, Signs and Symptoms	7464	22.5	3631	37.2	1775	53.2	1.65	2.36	1.43
Nervous System Diseases	5600	16.9	2753	28.2	1366	40.9	1.67	2.42	1.45
Congenital, Hereditary, and Neonatal Diseases and Abnormalities	5269	15.9	2599	26.6	1287	38.5	1.67	2.42	1.4
Digestive System Diseases	4710	14.2	2465	25.2	1253	37.5	1.77	2.64	1.49
Immune System Diseases	4583	13.8	2302	23.6	1170	35.0	1.70	2.53	1.49
Skin and Connective Tissue Diseases	4386	13.2	2256	23.1	1192	35.7	1.74	2.70	1.55
Urologic and Male Genital Diseases	4025	12.2	2103	21.5	1087	32.6	1.77	2.68	1.51
Endocrine System Diseases	4016	12.1	2060	21.1	1047	31.4	1.74	2.59	1.49
Cardiovascular Diseases	3969	12.0	2034	20.8	1031	30.9	1.74	2.58	1.48
Hemic and Lymphatic Diseases	3956	11.9	2049	21.0	1026	30.7	1.76	2.57	1.47
Female Genital Diseases and Pregnancy Complications	3826	11.6	2011	20.6	1028	30.8	1.78	2.66	1.50
Nutritional and Metabolic Diseases	3670	11.1	1897	19.4	955	28.6	1.75	2.58	1.47
Respiratory Tract Diseases	3524	10.6	1808	18.5	944	28.3	1.74	2.66	1.53
Musculoskeletal Diseases	3518	10.6	1751	17.9	913	27.3	1.69	2.57	1.53
Disorders of Environmental Origin	3466	10.5	1809	18.5	935	28.0	1.77	2.68	1.51
Virus Diseases	2963	8.9	1527	15.6	804	24.1	1.75	2.69	1.54
Mental Disorders	2903	8.8	1451	14.8	785	23.5	1.69	2.68	1.58
Bacterial Infections and Mycoses	2817	8.5	1441	14.7	754	22.6	1.73	2.65	1.53
Eye Diseases	2700	8.2	1318	13.5	709	21.2	1.65	2.60	1.57
Stomatognathic Diseases	2101	6.3	1069	10.9	583	17.5	1.72	2.75	1.60
Otorhinolaryngologic Diseases	1882	5.7	941	9.6	503	15.1	1.69	2.65	1.56
Parasitic Diseases	1739	5.3	918	9.4	493	14.8	1.79	2.81	1.57

Examples of MedGene analysis of disease term association with genes in PubMed, using either all human genes (2004), unique genes in MGC (2004), or HFLEX7000 (targets). Numerical values and percentiles of each class associated with genes are shown. Relative MeSH term associations in either MGC or HFLEX7000 to the genome, and in HFLEX7000 to MGC examine a potential bias in MGC or HFLEX7000 towards specific MeSH terms.

**Figure 2:**
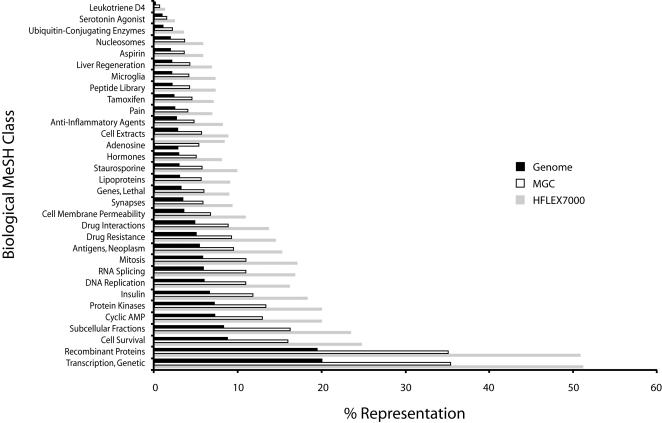
Genes associated with Biological MeSH Terms in Publications. The clone target list (HFLEX7000) was compared with all human genes (EntrezGene, 2004) and all genes represented by MGC (2004) with respect to published relationships of the genes to all biological MeSH terms and MeSH nodes (34). The targeted genes reveal similar proportionality to the other gene lists but a general enrichment of genes related to MeSH terms (Table 2; Supplementary Table S3).

In addition to disease relationships, we expanded our evaluation to include other search terms relevant for biological research by employing a new database, BioGene, which is based on a similar concept to MedGene. Instead of disease terms, BioGene has a co-citation index for all human genes with all biological and chemical Medical Subject Heading (MeSH) classes (http://www.nlm.nih.gov/mesh), such as “lipids”, “pain” and “tetrahydrofolates”, and is available at http://biogene.med.harvard.edu/BIOGENE/. As shown in Figure 2; Table 2 for 34 biological MeSH classes (complete listing for all analyzed MeSH terms in Supplementary Table S3), the candidate list is enriched for genes linked to all biological MeSH terms in the literature, but proportional to that of the entire MGC clones and to the entire genome.

**Table 2:** Biological and Chemical MeSH Class Analysis for Genes Associated in Publications with MeSH Class; Examples

Biological/Chemical MeSH Class	Genome	Genome	MGC	MGC	HFLEX7000	HFLEX7000	MGC/Genome	HFLEX 7000/MGC	HFLEX7000/Genome
	#	%	#	%	#	%			
Transcription, Genetic	6625	20.01	3457	35.38	1707	51.12	1.77	1.45	2.56
Recombinant Proteins	6454	19.49	3431	35.11	1697	50.82	1.80	1.45	2.61
Cell Survival	2912	8.79	1567	16.04	824	24.68	1.82	1.54	2.81
Subcellular Fractions	2753	8.31	1590	16.27	785	23.51	1.96	1.44	2.83
Cyclic AMP	2429	7.34	1271	13.01	670	20.07	1.77	1.54	2.74
Protein Kinases	2391	7.22	1307	13.37	668	20.01	1.85	1.50	2.77
Insulin	2212	6.68	1157	11.84	613	18.36	1.77	1.55	2.75
Mitosis	1917	5.79	1073	10.98	554	16.59	1.90	1.51	2.87
RNA Splicing	1950	5.89	1075	11.00	553	16.56	1.87	1.51	2.81
DNA Replication	1988	6.00	1071	10.96	540	16.17	1.83	1.48	2.69
Antigens, Neoplasm	1786	5.39	930	9.52	509	15.24	1.76	1.60	2.83
Drug Resistance	1645	4.97	910	9.31	486	14.56	1.87	1.56	2.93
Drug Interactions	1642	4.96	863	8.83	457	13.69	1.78	1.55	2.76
Cell Membrane Permeability	1195	3.61	669	6.85	365	10.93	1.90	1.60	3.03
Staurosporine	1012	3.06	570	5.83	331	9.91	1.91	1.70	3.24
Synapses	1128	3.41	581	5.95	312	9.34	1.75	1.57	2.74
Lipoproteins	1024	3.09	554	5.67	305	9.13	1.83	1.61	2.95
Genes, Lethal	1083	3.27	589	6.03	300	8.98	1.84	1.49	2.75
Cell Extracts	943	2.85	563	5.76	295	8.83	2.02	1.53	3.10
Adenosine	957	2.89	527	5.39	281	8.42	1.87	1.56	2.91
Hormones	976	2.95	499	5.11	271	8.12	1.73	1.59	2.75
Anti-Inflammatory Agents	891	2.69	473	4.84	270	8.09	1.80	1.67	3.01
Peptide Library	718	2.17	419	4.29	245	7.34	1.98	1.71	3.38
Microglia	700	2.11	406	4.15	241	7.22	1.97	1.74	3.41
Tamoxifen	776	2.34	445	4.55	237	7.10	1.94	1.56	3.03
Pain	812	2.45	391	4.00	229	6.86	1.63	1.71	2.80
Liver Regeneration	694	2.10	419	4.29	227	6.80	2.05	1.59	3.24
Aspirin	643	1.94	351	3.59	194	5.81	1.85	1.62	2.99
Nucleosomes	612	1.85	359	3.67	194	5.81	1.99	1.58	3.14

Examples of BioGene analysis of biological and chemical MeSH class associations with genes in PubMed, using either all human genes (2004), unique genes in MGC (2004), or HFLEX7000 (targets). Numerical values and percentiles of each class associated with genes are shown (#, %). Relative MeSH Class associations in either MGC or HFLEX7000 to the genome, and in HFLEX7000 to MGC examine a potential bias in MGC or HFLEX7000 towards specific MeSH terms.

Thus, the target set of 3557 genes had a similar overall distribution of genes as the MGC and the human genome, but in general has a higher representation of genes that have been linked to both diseases and biological terms in the literature.

### Clone Production and Sequence Validation

#### Production of Clone Collection

We generated the ORF clones via a processing pipeline that relies heavily on the use of robotics and is supported by the FLEXGene LIMS to produce clones in a highly automated, efficient, and accurate manner as published previously [5], [10], [16], [17].

**Figure 3:**
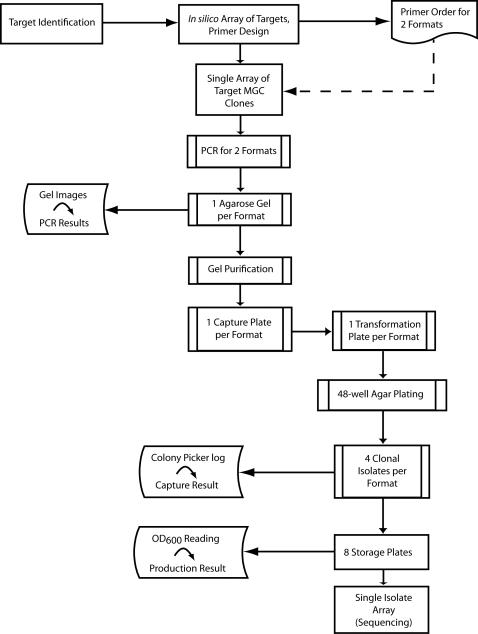
Workflow diagram of clone production. The entire production process from the design of primers to production of glycerol stocks is shown. The process started by identifying MGC clones in the available plates and then creating array files along with matching PCR primer order files that included two primers anchored at the 3′ end, one for each format. The primers were used to amplify the ORFs from the matching MGC clones. PCR products were monitored in agarose gels, and products were purified prior to capture via In-Fusion reaction. Competent bacterial strains were transformed with the reaction followed by the robotic isolation of 4 resulting colonies per format, which were used to prepare 15% glycerol stocks. Prior to sequencing a single isolate plate of 96 targets were created. As indicated, step specific results were stored in our LIMS.

The process of converting MGC cDNA clones into ORF clones (Figure 3) was initiated by populating our production tracking database (FLEXGene) with the relevant MGC information, e.g. IMAGE ID, GI number, clone sequence, start/stop of CDS, CDS length, gene information, plate and position in IMAGE/MGC collection. All ORFs were normalized to start with ATG, and natural stop codons either to TAG, or, for the format without C-terminal stop, to TTG (Leu). PCR amplicons were gel purified and captured using the In-Fusion™ enzyme into a modified recombinational cloning vector, pGWNcoXho, which increased the efficiency of capturing DNA fragments larger than 1.5 kb [16]. After transformation into *E. coli*, constructs were clonally selected and isolated. In total, we successfully produced clonal glycerol stocks for 3,528 of 3,557 targeted genes, an overall success rate of ∼98% (Table 3).

**Table 3:** Clone Production and Sequencing Summary

	Type	Phase1 (1st isolate)	Phase2 (2nd isolate)	Total
ORF Target	CLOSED	3557	277	3557
	FUSION	3557	327	3557
Avg ORF size and range (bp)	ALL	1268 (99–4,785)	1491 (171–4,395)	1268 (99–4,785)
Clones for sequence validation	CLOSED	3535	277	3812
	FUSION	3496	327	3823
Number of reads	ALL	20672	1957	22629
Average number of reads per clone	ALL	2.9	3.2	2.9
Accepted clones	CLOSED	3240 (91%)	223 (81%)	3463 (91%)
	FUSION	3242 (92%)	258 (79%)	3500 (92%)
	ALL	6482	481	6963
Accepted clone match perfect with reference	ALL	6226	443	6669 (95.8%)
Accepted clone with silent mutation(s) only	ALL	94	13	107 (1.5%)
Accepted clone with 1 mis-sense	ALL	162	25	187 (2.7%)
Rejected clones	ALL	646 (9%)	119 (20%)	765 (10%)
Clones rejected for linker changes	ALL	158	43	201 (26%)
Clones rejected for cds changes (ins/del/nonsense/mis)	ALL	187	48	235 (31%)
Clones rejected for no/incomplete/wrong assembly	ALL	301	28	329 (43%)
Accepted ORFs	CLOSED	3097 (88%)	222 (80%)	3295 (93%)
	FUSION	3075 (88%)	256 (78%)	3259 (92%)
	ALL	3387	453	3447 (97%)

#### DNA sequence analysis and clone acceptance

Based on a pilot study of 96 genes in which we sequenced all available isolates (4 closed and 4 fusion), we expected that 90% of the clones would yield a valid clone. Thus, it was most efficient to sequence a single isolate for each attempted clone format and return to evaluate additional isolates for any that failed. Clones were accepted if they had no truncation mutations, no frameshift mutations and no more than one single amino acid difference with the reference sequence. Clones with any nucleotide changes in the att- sequences were rejected, as changes in these regions could make it impossible to transfer the ORF into expression vectors (Table 3).

All rejected clones were manually inspected including a BLAST-based comparison to GenBank/EMBL to assess whether the clone matched any other entry for this gene. This step helped to rescue some rejected clones which were found to be ultimately acceptable due to updated MGC sequence entries.

Consistent with the pilot study, 6963 (90%) of the sequenced clones were acceptable based on the above criteria, with the vast majority (6669, or 95.8%) matching exactly to the reference sequence (Table 3, for complete listing of clones see Supplementary Table S4). There were 25 clones that had identical discrepancies in both formats (with and without stop codon). As the two formats were independently produced from the same source clone, this suggests that there may be mistakes in as many as 0.3% of MGC reference sequences.

## Discussion

Starting from the MGC resource, we created protein expression ORF clones in two different formats for over 3400 human genes, HFLEX7000, making them the largest contribution of fully sequence verified ORF clones to the ORFeome Collaboration (www.Orfeomecollaboration.org ). The selection criteria for this subset were based on a combination of publication records for the individual gene and their association with biological as well as human disease MeSH terms, as defined by two programs, MedGene and BioGene. We aimed to reflect within this subset a similar distribution as it was present in MGC or the genome, and not to create a functionally or disease specific subset.

To assure the quality of this cDNA clone collection, we fully sequence verified all clones. By employing the appropriate formatted clone, users can add peptide tags to either end of the expressed protein or express protein without any additional amino acids at all. This is important for application reasons, e.g., for some proteins, the C-terminal amino acids may be important functionally (PDZ domain [18]) requiring a translation stop at the natural position, whereas for other proteins the natural N-terminus is relevant (e.g., signal peptides for membrane protein trafficking [19], [20]). Some applications exploit the use of fusion tags at the C-terminus as an experimental readout (e.g., yeast two hybrid [7]), or for capturing expressed proteins and confirming full length expression [21].

We targeted over 3500 unique genes and obtained a fully sequence validated ORF clone for 97% (>3400) of the genes. The strategy of selecting only one clonal isolate per gene for sequencing successfully yielded 90% acceptable clones. This success rate dropped to 80% when second isolates of the failed clones were sequenced, raising questions about the likelihood of success of sequencing additional isolates for clones that failed after two attempts. Also, capture efficiency, as measured by the number of colonies after transformation, was not a predictor of eventual clone success; clones with either high or low colony count numbers were equally likely to be rejected at subsequent steps.

One set of troublesome ORFs identified during PCR and confirmed during sequencing revealed duplication of either the 5′ (near the ATG) or 3′ (near the stop codon) sequences used to design the PCR primer elsewhere in the clone. This led to inappropriate PCR priming and ultimately an inability to clone the gene. Any project using a similar strategy to convert MGC clones into ORF clones might find the same problems, and alternatives, e.g. restriction enzyme/ligation based or fragmented PCR cloning, should be considered for any such ORFs.

In summary, the clones from MGC provide an excellent resource for ORF clone production. The 97% success rate to produce fully sequence validated clones, of which 96% match perfectly to the template clone, underlines that this strategy is feasible in a cost effective manner. Together with our other human ORF sets, notably several hundred DNA binding proteins, over 500 kinases, 1000 breast cancer associated genes, this much broader collection of 3500 genes will be of great benefit to the research community.

As with all our sequence validated clone collections, human, yeast, or microorganism, the HFLEX7000 clones are available at http://plasmid.hms.harvard.edu. Furthermore, this collection as part of the global ORFeome Collaboration will be available from the ORFeome distributors (http://www.orfeomecollaboration.org/).

## Materials and Methods

### ORF-specific primer design and strategic 96-well plate organization

ORF sequences were parsed out in the FLEXGene LIMS from the information provided with each MGC clone, and primer sequences were designed using a nearest neighbor algorithm as described earlier [5], [10], [16], [17]. Natural stop codons were either normalized to TAG or replaced with TTG (Leu) in the final primer designs. In addition to ORF-specific, start and stop regions, the 5′ and 3′ primers included fixed sequences that correspond to partial att sequence-specific recombination recognition sites that flank the ORF in the resultant plasmid clones.

Details regarding amplification, purification and capture into a linear version of pDONR221 by In-fusion™ reaction were previously published [16]; PCR success as measured as signal in agarose gels, and capture success as determined as colonies after transformation were stored in FLEXGene LIMS as reported elsewhere [10], [16], [17].

### Clone isolation and production of glycerol stocks

Transformations into *E. coli* (DH5alpha, T1 resistant) were handled in 96-well plates, and robotically plated to 48-sector LB/agar dishes with the appropriate antibiotic selection and grown overnight at 37°C. Colonies were robotically visualized and counted, and single isolates from each sector were picked for inoculation into 1mL growth media (LB/antibiotic) using a customized Megapix robot (Genetix), and 96-well culture blocks were grown overnight at 37°C in the presence of appropriate antibiotic. Inoculated cultures were assayed for growth via OD_600_ measurement as a measure of transformation efficiency, and aliquots were stored as 15% glycerol stocks in 96-well plate format.

### Sequence reactions

High-throughput sequencing was carried out on an Applied Biosystems (ABI) capillary sequencer using dye-terminator and fluorescent cycle sequencing with don3 (TCTTGTGCAATGTAACATCAG) and don5 (CGTTAACGCTAGCATGGA) primers. Raw sequence data were automatically analyzed for quality, vector and repeat content using the pregap4 tool of the Staden Software Package [22]. Reads passing this initial quality control were automatically assembled (gap4 tool of the Staden Software Package). The primer walking method was used to finish insert sequencing, with primers automatically designed by PRIDE [23]. Sequencing was finished when an overall sequence quality of phred40 for the insert sequence and the vector-insert transition was achieved.

### Sequence Analysis Software

After the clone sequence was assembled in the Staten package, the assembled sequences were verified using in house developed software [24]. Clones with acceptable linker as well as CDS sequences were collected for distribution. Clones were not accepted if they had discrepancies leading to protein truncations, frame shifts, discrepancies in the linker regions, or more than two amino acid differences with the reference polypeptide. Sequences of all clones started and ended at the BsrGI restriction site (TGTACA) of the vector, allowing QC of in-frame analysis as well as intactness of att recombination sites. Only clones that failed the CDS region evaluation underwent BLAST search against all available GenBank records, and were re-evaluated using matching BLAST hits in pairwise alignments, allowing us to rescue ∼5% of the clones.

## Supporting Information

Table S1 Target Gene List for HFLEX7000. MGC clones targeted for conversion into ORF clones are listed with Gene Symbol, Entrez GeneID, gene name, GenBank Acc. No., and CDS Length
(0.62 MB XLS)

Table S2 Disease MeSH Term Analysis. Complete Analysis for Gene and Diseases Association in Publications
(0.57 MB XLS)

Table S3 Biological and Chemical MeSH Class Analysis. Complete BioGene analysis of biological and chemical MeSH class associations with genes in PubMed, using either all human genes (2004), unique genes in MGC (2004), or HFLEX7000 (targets). Numerical values and percentiles of each class associated with genes are shown (#, %). Relative MeSH Class associations in either MGC or HFLEX7000 to the genome, and in HFLEX7000 to MGC examine a potential bias in MGC or HFLEX7000 towards specific MeSH terms.
(1.87 MB XLS)

Table S4 HFLEX7000 Clone List. Complete listing of all accepted, fully sequence validated ORF clones with Gene Symbol, Entrez GeneID, PlasmID CloneID, Clone GenBank Acc. No., Clone IMAGE ID, and Reference GenBank Acc. No.
(1.20 MB XLS)
